# Supplementary material for: Comparative multiomics analysis of cell physiological state after culture in a basket bioreactor
Source: Sci Rep. 2022 Nov 23;12:20161. doi: 10.1038/s41598-022-24687-4 (PMC9686226; doi:10.1038/s41598-022-24687-4)
Supplement: Supplementary file 1 — Supplementary Information 1. [file 41598_2022_24687_MOESM1_ESM.zip › raw data/Transcriptomic raw data/8.SNP/3.stat/INDEL_region.pdf]

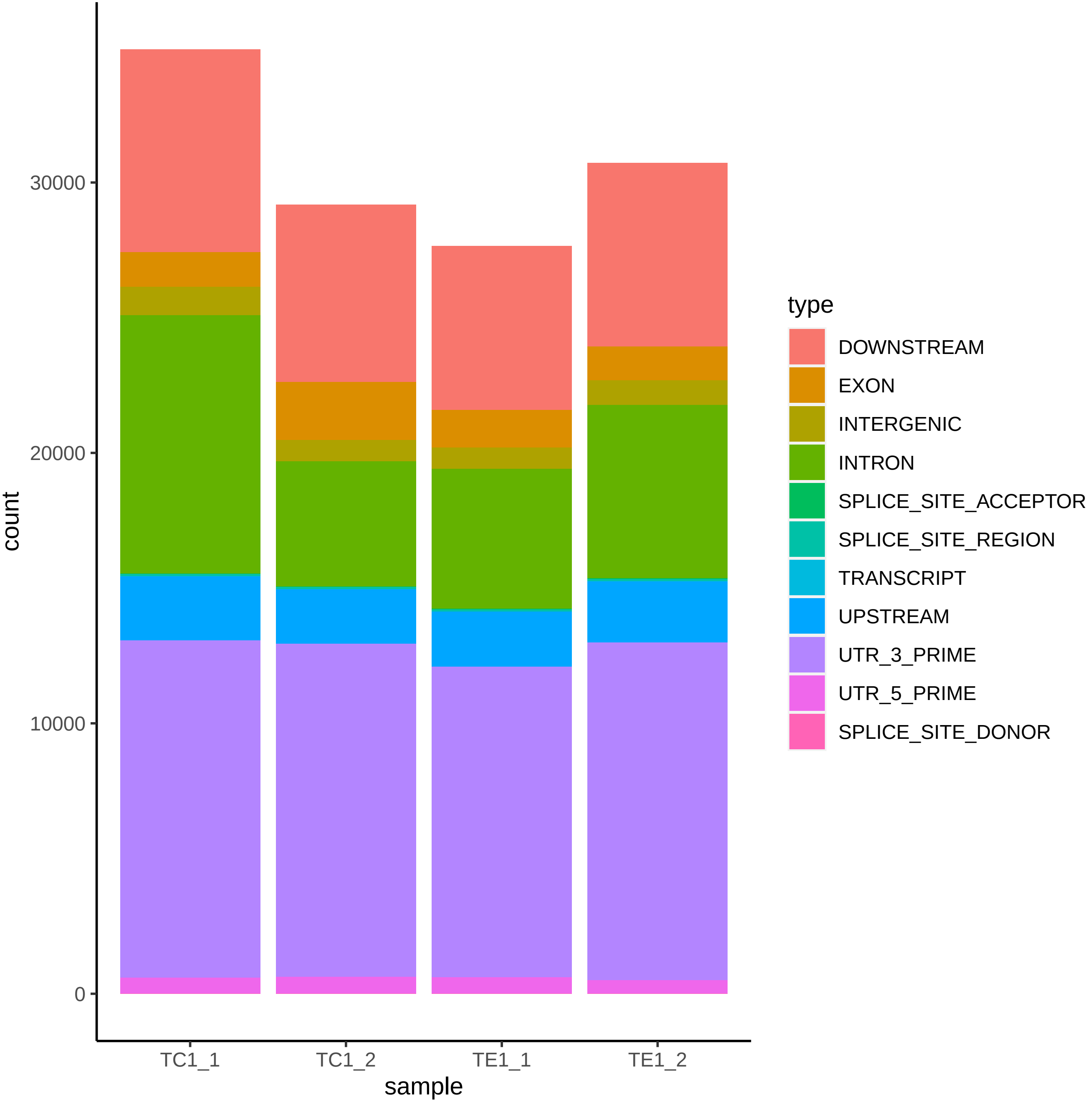

30000
20000
10000
0
count
TC1_1
TC1_2
TE1_1
TE1_2
sample
type
DOWNSTREAM
EXON
INTERGENIC
INTRON
SPLICE_SITE_ACCEPTOR
SPLICE_SITE_REGION
TRANSCRIPT
UPSTREAM
UTR_3_PRIME
UTR_5_PRIME
SPLICE_SITE_DONOR
